# Commentary: Adenovirus and *Mycoplasma pneumoniae* co-infection as a risk factor for severe community-acquired pneumonia in children

**DOI:** 10.3389/fped.2024.1464813

**Published:** 2024-12-02

**Authors:** Duc Long Phi, Minh Manh To, Kieu Dung Le, Tien Dat Pham, Cao Thanh Vu, Khanh Linh Duong, Thi Loi Dao, Van Thuan Hoang

**Affiliations:** Thai Binh University of Medicine and Pharmacy, Thai Binh, Vietnam

**Keywords:** *Mycoplasma pneumoniae*, pneumonia, children, risk factors, adenovirus

A Commentary on Adenovirus and *Mycoplasma pneumoniae* co-infection as a risk factor for severe community-acquired pneumonia in children By Chen Q, Lin L, Zhang N and Yang Y (2024). Front. Pediatr. 12.1337786. doi:10.3389/fped.2024.1337786

## 1 Introduction

We have read with great interest the publication by Chen et al. (1) on the co-infection of adenovirus and *Mycoplasma pneumoniae* among children with severe community-acquired pneumonia (CAP). We especially appreciate the findings about risk factors of severe disease. Several studies have showed whether *M. pneumoniae* infection contributes to more severe clinical outcomes than other pathogens (2–5). However, the role of *M. pneumoniae* shoud be considered along with other factors such as age, gender, and co-infection with different pathogens. In this Commentary, we report no association between *M. pneumoniae,* but an association with age, and severe pneumonia in children.

*M. pneumoniae* is a common cause of respiratory infections, particularly in children and young adults. Despite its prevalence, the role of *M. pneumoniae* in the disease severity among children remains controversial (6–9). As a bacterium without a cell wall, *M. pneumoniae* spreads via respiratory droplets, causing mild, self-limiting infections. It is common in school-aged children and can cause outbreaks in community settings (10, 11).

*M. pneumoniae* accounts for 10%–40% of pediatric CAP cases with peaks in children aged 5 to 15. Epidemiological studies suggest cyclical outbreaks of *M. pneumoniae* infections every 4–7 years, with regional and seasonal variation. Severe cases are more common in children with underlying health conditions (12). Research findings on the link between *M. pneumoniae* and severe pneumonia have been mixed, as summarized in a recent systematic review (9).

## 2 No association between *M. pneumoniae* infection and severe pneumonia among children

From 01/06/2023 to 31/05/2024, 2040 children with atypical pneumonia were hospitalized at Thai Binh Pediatric Hospital, Thai Binh. The proportion of severe pneumonia was 11.9% (243/2040). Due to limitations in equipment and the fact that health insurance does not cover molecular biological tests for diagnosing respiratory pathogens, the identification of *M. pneumoniae* infection in children in Thai Binh is only performed using serological methods. The serum samples were quantitatively analyzed for IgM antibodies against *Mycoplasma pneumoniae* using the Virion/Serion ELISA kit (GmbH Germany, catalog number ESR127M). The antibody levels were expressed in units per milliliter (U/ml). According to the manufacturer's guidelines, the interpretation criteria for *M. pneumoniae* IgM were as follows: positive (>17 U/ml), negative (<13 U/ml), and borderline (13–17 U/ml). All ELISA assays were carried out strictly following the instructions provided by the manufacturer. A total of 1070 (52.4%) patients were positive with IgM. The distribution of ill children gradually decreased with age.

Univariate and multivariate analysis using logistic regression showed no association between *M. pneumoniae* infection and male gender with severe pneumonia among children (adjusted OR = 0.98, *p* = 0.91, and aOR = 1.21, *p* = 0.18, respectively). However, age was a risk factor closely related to the severity of pneumonia. Compared to children aged ≥5 years old, those aged 2–<12 months were associated with 7 times of risk for severe disease (aOR = 6.87, *p* < 0.0001), patients aged 12–<36 months and 36–<60 months were associated with 4 times and twice of risk for severe pneumonia (aOR = 3.39, *p* < 0.0001 and aOR = 1.84, *p* = 0.002, respectively) (Table 1).

**Table 1:** Risk factors of atypical pneumoniae among children (*N* = 2,040).

Characteristics	Total	Non-severe pneumonia	Severe pneumonia	Univariate analysis	Multivariate analysis
	*n* (%)	*n* (%)	*n* (%)	OR [95%CI] *p*-value	adjusted OR [95%CI] *p*-value
Age					
≥5 years	744 (36.4)	698 (38.8)	46 (18.9)	Reference	Reference
36–<60 months	644 (31.6)	574 (31.9)	70 (28.8)	1.85 [1.26–2.73] 0.002	1.84 [1.25–2.72] 0.002
12–<36 months	605 (29.7)	493 (27.4)	112 (46.1)	3.45 [2.40–4.95] <0.0001	3.39 [2.34–4.90] <0.0001
2–<12 months	47 (2.3)	32 (1.8)	15 (6.2)	7.11 [3.60–14.07] <0.0001	6.87 [3.41–13.84] <0.0001
<2 months	0 (0)	0 (0)	0 (0)	–	–
Gender					
Female	881 (43.2)	790 (44.0)	91 (37.4)	Reference	Reference
Male	1,159 (56.8)	1,007 (56.0)	152 (62.6)	1.31 [0.99–1.73] 0.06	1.21 [0.92–1.61] 0.18
*Mycoplasma pneumoniae* IgM					
Negative	970 (47.6)	839 (46.7)	131 (53.9)	Reference	Reference
Positive	1,070 (52.4)	958 (53.3)	112 (46.1)	0.75 [0.57–0.98] 0.04	0.98 [0.74–1.31] 0.91

## 3 Discussion

The heterogeneity in study designs, populations, and diagnostic methods contributes to the variability in the results assessing the role of *M. pneumoniae* in severe pediatric pneumonia (6).

One of the primary challenges in establishing a clear association is the presence of confounding factors. Children with severe pneumonia are more likely to undergo extensive diagnostic testing, including polymerase chain reaction (PCR) assays, leading to a detection bias. Additionally, co-infections with other respiratory pathogens, such as viruses or bacteria, can complicate the clinical picture and obscure the role of *M. pneumoniae*.

The lack of a consistent association between *M. pneumoniae* infection and severity of disease has important implications for clinical practice (7, 8). Physicians should consider *M. pneumoniae* a potential cause of pneumonia in children but not assume it is associated with more severe disease without supporting clinical evidence. Treatment decisions should be guided by the overall clinical picture and not solely based on the presence of *M. pneumoniae*.

Indeed, antibiotic therapy for *M. pneumoniae* pneumonia typically includes macrolides, such as azithromycin or clarithromycin. However, the emergence of macrolide-resistant *M. pneumoniae* strains poses a challenge (13, 14). Clinicians must remain vigilant for resistance patterns and consider alternative treatments in older children, such as fluoroquinolones or tetracyclines.

Further research is needed to clarify the relationship between *M. pneumoniae* and pneumonia severity in children. Prospective studies with standardized diagnostic criteria, robust case definitions, and comprehensive data collection on co-infections and underlying health conditions are essential.

It is also crucial to understand the mechanisms underlying macrolide resistance in *M. pneumoniae* and develop strategies to minimize its impact. Surveillance programs to monitor resistance patterns and the effectiveness of current treatment regimens can inform clinical guidelines and improve patient outcomes.

The findings of our study should be interpreted with caution due to several limitations. Firstly, although the large sample size, this was a retrospective study conducted at a single center, focusing only on the association of severe pneumonia with age, sex, and *M. pneumoniae* infection. Other potential factors that might influence the severity of pneumonia in children, such as underlying health conditions, socioeconomic factors, or exposure to environmental pollutants, were not assessed. Including these variables in future research would provide a more comprehensive understanding of the risk factors associated with severe pneumonia in pediatric patients. Furthermore, due to limited laboratory resources, *M. pneumoniae* infection was identified using a single IgM test, whereas paired sera would provide a more accurate diagnosis. The use of paired sera, with samples collected at least two to four weeks apart, is widely regarded as the preferred approach for confirming M. pneumoniae infection, as it helps to distinguish between acute infection and past exposure. However, the reliability of single IgM serological tests and the requirement for paired testing remain inconsistent (15). However, implementing paired testing poses logistical challenges, especially in children, as it requires additional blood samples over time. Additionally, while paired IgG testing is often used as a reference standard, its relevance to routine clinical practice and its impact on treatment decisions remain limited, especially in pediatric settings.

In conclusion, the current body of evidence does not support a definitive association between *M. pneumoniae* infection and the severity of pneumonia among children. The variability in study findings highlights the complexity of pneumonia etiology and the influence of multiple factors on disease severity. Clinicians should adopt a holistic approach to diagnosing and managing pediatric pneumonia, considering the potential role of *M. pneumoniae* without overestimating its impact on disease severity.
